# TRIF-dependent signaling and its role in liver diseases

**DOI:** 10.3389/fcell.2024.1370042

**Published:** 2024-04-17

**Authors:** Lilin Hu, Zilu Cheng, Huikuan Chu, Weijun Wang, Yu Jin, Ling Yang

**Affiliations:** Division of Gastroenterology, Union Hospital, Tongji Medical College, Huazhong University of Science and Technology, Wuhan, China

**Keywords:** TRIF, TLR3, TLR4, IFN-β response, NF-κB, apoptosis, necroptosis, liver diseases

## Abstract

TIR domain-containing adaptor inducing IFN-β (TRIF) is a crucial adaptor molecule downstream of toll-like receptors 3 (TLR3) and 4 (TLR4). TRIF directly binds to TLR3 through its TIR domain, while it associates with TLR4 indirectly through the bridge adaptor molecule TRIF-related adaptor molecule (TRAM). TRIF plays a pivotal role in regulating interferon beta 1 (IFN-β) response, nuclear factor kappa B (NF-κB) signaling, apoptosis, and necroptosis signaling mediated by TLR3 and TLR4. It accomplishes these by recruiting and activating various kinases or transcription factors via its distinct domains. In this review, we comprehensively summarize the TRIF-dependent signaling pathways mediated by TLR3 and TLR4, elucidating key target molecules and downstream pathways. Furthermore, we provide an overview of TRIF’s impact on several liver disorders, including drug-induced liver injury, ischemia-reperfusion liver injury, autoimmune hepatitis, viral hepatitis, alcohol-associated liver disease (ALD), metabolic dysfunction-associated steatotic liver disease (MASLD) and metabolic dysfunction-associated steatohepatitis (MASH). We also explore its effects on liver steatosis, inflammation, fibrosis, and carcinogenesis. A comprehensive understanding of the TRIF-dependent signaling pathways, as well as the intricate relationship between TRIF and liver diseases, can facilitate the identification of potential drug targets and the development of novel and effective therapeutics against hepatic disorders.

## 1 Introduction

The human body utilizes pattern recognition receptors (PRRs) to detect specific molecular signatures of invading pathogens, initiating innate immune responses (Sato et al., 2003; Kong et al., 2021; Dibo et al., 2022). This recognition system not only aids in the elimination of encountered microorganisms but also plays a pivotal role in adaptive immune responses, mainly by inducing the release of inflammatory cytokines and facilitating the expression of co-stimulatory molecules on antigen-presenting cells (APCs) (Kawai et al., 2001). Toll-like receptors (TLRs), the first identified PRRs, are crucial for antigen presentation, host defense, and the regulation of cell death (Xun et al., 2021; Duan et al., 2022; Garantziotis and Savani, 2022). To date, 10 functional TLRs have been identified in humans and 12 in mice (Luo et al., 2019). Strategically distributed on both the cell surface and within the cell, TLRs provide a comprehensive immune defense. Structurally, TLRs consist of three parts: an extracellular leucine-rich repeat region, a transmembrane region, and a cytoplasmic tail structure (Yang et al., 2022). The cytoplasmic tail, also known as Toll-interleukin-1 receptor homologous domain (TIR domain) is critical for each TLR’s recognition of different ligands through its extracellular leucine-rich repeats. Subsequently, the cytoplasmic TIR domain mediates the interaction between TLRs and their downstream TIR-domain-containing adaptor molecules, triggering a series of pathways to induce downstream biological functions (Kaisho and Akira, 2006).

TIR domain-containing adaptor inducing IFN-β (TRIF), also known as TIR-containing adaptor molecule-1 (TICAM-1), is an adaptor molecule downstream of TLR3 and TLR4 (Oshiumi et al., 2003; Sato et al., 2003). TRIF directly binds to the intracellular TIR domain of TLR3 via its own TIR domain, making it the sole adaptor protein with this capability (Oshiumi et al., 2003; Meylan et al., 2004). In contrast, TLR4 signaling can be mediated by both TRIF and Myeloid differentiation 88 (MyD88). TRIF binds to TLR4 indirectly through the bridge molecule TRIF-related adaptor molecule (TRAM) (He et al., 2011). TLR4 activation occurs in response to lipopolysaccharide (LPS) derived from gram-negative bacteria. TLR3, localized on the membrane of the endoplasmic reticulum, endosome, or lysosome, is activated by double-stranded RNA (dsRNA) from viruses or synthetic dsRNA mimics such as poly (I: C) (He et al., 2011). Activation of TLR3/TLR4 triggers intracellular signaling pathways, leading to nuclear translocations of downstream transcription factors, the activation of mitogen-activated protein kinases (MAPK) pathways, interferon beta 1(IFN-β) response, and the nuclear factor kappa B (NF-κB) signaling pathway (Meylan et al., 2004). Additionally, TLR3/TLR4 plays a role in modulating cell death signaling pathways, including apoptosis and necroptosis (Kalai et al., 2002; Ma et al., 2005).

**FIGURE 1:**
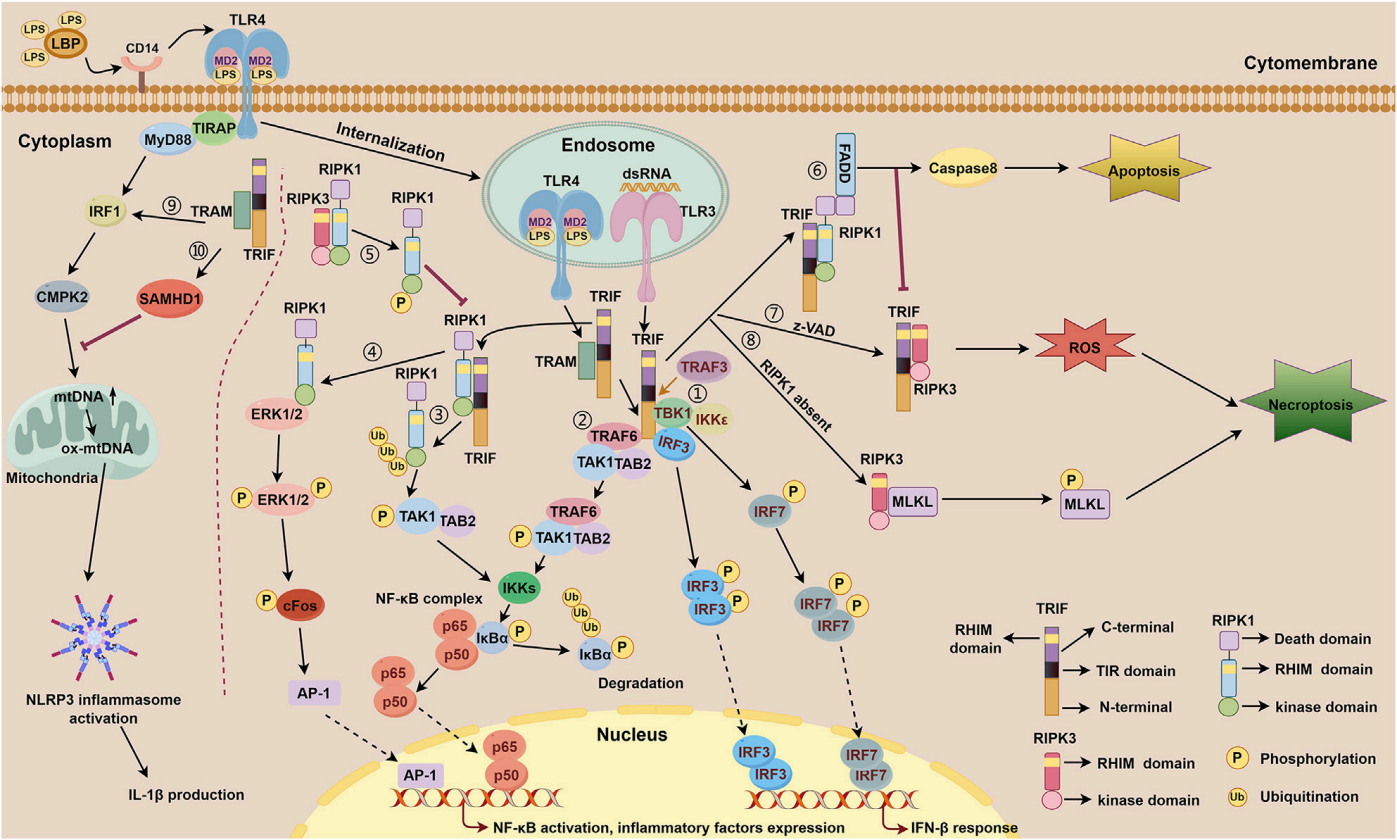
TRIF-dependent TLR3 and TLR4 signaling. TLR4 forms a complex with MD-2, a secreted glycoprotein that recognizes and binds LPS through the participation of LBP and CD14. Subsequently, TLR4 is internalized to the membrane of the endosome and relies on bridge adaptor TRAM to initiate TRIF-dependent signaling. TLR3 is localized on the membrane of the endosome and activated by dsRNA, which interacts with TRIF directly. TRIF-dependent TLR3 signaling includes ①-⑧. TRIF-dependent TLR4 signaling includes ①-⑩. ① IFN-β response mediated by the N-terminal of TRIF downstream of TLR3 and TLR4. Upon activation of the TRAF3, the N-terminal of TRIF interacts with TBK1 and IKKε. TBK1 phosphorylates TRIF and then facilitates IRF3 binding to TRIF. Consequently, TBK1 phosphorylates IRF3 and IRF7, facilitating the formation of phosphorylated dimers for both proteins. These dimers subsequently translocate into the nucleus to initiate activation of the IFN-β response. ② NF-κB signaling mediated by the N-terminal of TRIF downstream of TLR3 and TLR4. TRIF recruits TRAF6, which then binds to TAK1 and TAB2 to activate IKKs. Subsequently, activated IKKs phosphorylate IκBα, thereby abolishing its inhibitory function on NF-κB complex and facilitating its activation. ③ NF-κB signaling mediated by the C-terminal of TRIF downstream of TLR3 and TLR4. The C-terminal of TRIF interacts with RIPK1 via their RHIM domains, followed by polyubiquitination events of RIPK1 which then facilitates the combination of TAK1 and TAB2, subsequently promoting IKKs activation, IκBα phosphorylatin, and NF-κB activation. ④ NF-κB signaling mediated by the C-terminal of TRIF downstream of TLR4. The C-terminal of TRIF interacts with RIPK1 through their RHIM domains, facilitating direct binding of RIPK1 to ERK1/2. Subsequently, phosphorylation of ERK1/2 occurs, inducing AP-1 activation and ultimately resulting in the expression of inflammatory factors. ⑤ RIPK3 competitively inhibits TRIF-dependent TLR3/TLR4-RIPK1-NF-κB signaling via its RHIM domain. RIPK3 interacts with RIPK1 through their RHIM domains. This interaction leads to the phosphorylation of RIPK1, thereby inhibiting RIPK1-dependent NF-κB activation processes. ⑥ Apoptosis signaling mediated by the RHIM domain of TRIF downstream of TLR3 and TLR4. The RHIM domain of TRIF allows it to bind to RIPK1. Once bound, RIPK1 recruits FADD through its DD domain, resulting in the activation of caspase8 and subsequently inducing apoptosis. ⑦-⑧ Necroptosis signaling mediated by the RHIM domain of TRIF downstream of TLR3 and TLR4. ⑦ In the presence of z-VAD, TRIF interacts with RIPK3 via their RHIM domains, then facilitates the accumulation of ROS, leading to the initiation of necroptosis. ⑧ When RIPK1 is absent, TRIF promotes the interaction between RIPK3 and MLKL, resulting in the phosphorylation of MLKL, thereby triggering necroptosis. During these processes, the FADD-caspase8 complex inhibits the activation of RIPK3 to restrain necroptosis. ⑨ NLRP3 inflammasome activation and mtDNA synthesis mediated by MyD88 and TRIF downstream of TLR4. MyD88 and TRIF trigger the transcription of CMPK2 with the involvement of IRF1, promoting the synthesis of mtDNA and ox-mtDNA, ultimately resulting in the activation of NLRP3 inflammasome. ⑩ TRIF activates the expression of SAMHD1 which exhibits a negative feedback role in mtDNA synthesis and IL-1β production.

TRIF is a versatile adaptor protein comprising a TIR domain flanked by N-terminal and C-terminal extensions (Ve et al., 2012). The N-terminal region recruits various kinases and transcription factors, including TANK binding kinase 1 (TBK1) and TNF receptor-associated factor 6 (TRAF6). These interactions induce an IFN-β response and activate the NF-κB signaling pathway, respectively (Yamamoto et al., 2002; Sato et al., 2003). The C-terminal region of TRIF also contributes to NF-κB signaling pathway activation and plays a vital role in regulating cell apoptosis and necroptosis (Ullah et al., 2016). Protein structural sequence analysis has revealed a 35 amino acid fragments in the C-terminal region of TRIF known as RIP homotypic interaction motif (RHIM). It is now understood that TRIF interacts with receptor-interacting protein 1 (RIPK1) and receptor-interacting protein 3 (RIPK3) through its RHIM domain, participating in tumor necrosis factor (TNF)-induced NF-κB activation (Meylan et al., 2004). TRIF’s central role in TLR signaling necessitates a comprehensive overview of TRIF-dependent TLR signaling pathways. The following sections provide detailed descriptions of the TRIF-mediated signaling pathways initiated by TLR3 and TLR4 (summarized in Table 1; Figure 1).

**TABLE 1:** TRIF-dependent TLR3 and TLR4 signaling.

References	Cell types	Signaling molecules and pathways	Biological functions
Fitzgerald et al. (2003)	293T cells	TLR3-TRIF- IKKε/TBK1-IRF3	IRF3 activation
Sato et al. (2003)	HEK293 cells	TRIF-TBK1-IRF3-IFN-β	IFN-β response
	HEK293/Mouse Peritoneal macrophages	TLR3/TLR4-TRIF-TRAF6-NF-κB	NF-κB activation
Jiang et al. (2004)	HEK293 cells/HeLa cells	TLR3-TRIF-TRAF6-TAK1-TAB2-NF-κB	NF-κB activation
Cusson-Hermance et al. (2005)	MEFs	TLR3/TLR4-TRIF-TRAF6-IκBα-NF-κB	NF-κB activation
	MEF/splenocytes/HEK293T cells	TLR4-TRIF-RIPK1-NF-κB	NF-κB activation
Meylan et al. (2004)	MEFs	TLR3-TRIF-RIPK1-NF-κB	NF-κB activation
	293T cells	TLR3-TRIF-RIPK3-NF-κB	Inhibiting NF-κB activation
McAllister et al. (2013)	Small intestinal mucosal epithelial cells	TLR3-TRIF-Caspase8-apoptosis	Mucosal remodeling and recovery
Yu et al. (2011)	NCI-H292	TLR3-TRIF-RIPK1-Caspase-apoptosis	sTNFR1 release
He et al. (2011)	BMDMs	TLR3/TLR4-TRIF-RIPK3-ROS	Necroptosis
Cook et al. (2014)	MEFs	TLR3-TRIF-RIPK3-MLKL-necroptosis	Necroptosis
Tsukamoto et al. (2018)	BMDMs/RAW264 cells	TLR4-TRIF-TBK1-IKKε-IRF3-IFN-β	IFN-β response
Najjar et al. (2016)	BMDMs	TLR4-TRIF-RIPK1-ERK1/2-cFos	Inflammatory genes expression
Kaiser and Offermann (2005)	HEK293 cells	TRIF-RIPK1-FADD-Caspase8	Apoptosis
Zhong et al. (2018)	Macrophage	TLR4-TRIF-IRF1-CMPK2	Promoting mtDNA synthesis and NLRP3 activation
		TLR4-TRIF-SAMHD1	Inhibiting mtDNA synthesis and IL-1β production

Abbreviations: HEK, human embryonic kidney; MEFs, Mouse embryonic fibroblasts; BMDMs, Bone marrow derived macrophages.

TRIF-dependent TLRs play distinct roles in different systems, with effects associated with specific tissues and cellular environments. Previous studies have demonstrated the cardioprotective and neuroprotective effects of TLR3-TRIF signaling (Riad et al., 2011; Reinert et al., 2012). However, in respiratory viral infections, the TLR3-TRIF pathway can be detrimental by triggering excessive inflammation (Le Goffic et al., 2006; Hutchens et al., 2008). In liver viral infection, TLR3/TRIF-mediated signaling acts as a double-edged sword (Li et al., 2012; Ayoobi et al., 2013). Additionally, TLR4-TRIF signaling has protective functions in respiratory and intestinal bacterial infections (Jeyaseelan et al., 2007; Cai et al., 2009; Gurung et al., 2012; Sotolongo et al., 2012), as well as roles in arthritis and tissue repair (Yarilina et al., 2007; Lin et al., 2012). With the global rise of liver diseases, the impact of the TRIF molecule in diverse liver pathologies has gained significant momentum, including viral hepatitis, metabolic dysfunction-associated steatohepatitis (MASH), alcoholic hepatitis, and more. These diseases often manifest as hepatocyte steatosis, inflammation, fibrosis, and other related processes. Furthermore, considering the crucial roles of immune response and cell death signals in liver diseases, we summarize the role of TRIF in the aforementioned hepatic pathological processes.

## 2 TRIF-dependent TLR3 signaling

TLR3 and TLR4 are indispensable components of the immune system, playing a crucial role in recognizing pathogen-associated molecular patterns (PAMPs) and initiating intricate signaling cascades. Both receptors employ TRIF as a mediator to activate NF-κB, a transcription factor with pivotal roles in regulating the immune response, inflammation, and cell survival. TRIF is absolutely essential for TLR3-mediated NF-κB activation; its absence effectively inhibited this pathway (Yamamoto et al., 2003). Furthermore, TRIF participates in the IFN-β response and cell death signaling pathway triggered by TLR3, demonstrating its versatility as an adaptor molecule. The NF-κB signaling and IFN response induced by TLR3 primarily serve as an innate defense mechanism against viral infections, providing a robust virus recognition system. TLR3 specifically recognizes viral double-stranded RNA, triggering an antiviral immune response (Meylan et al., 2004). TLR4 primarily triggers NF-κB signaling and IFN response through the recognition of lipopolysaccharide (LPS) or endogenous damage-associated molecular patterns (DAMPs) (Brubaker et al., 2015). The IFN response mediated by TLR3 and TLR4 share fundamental similarities. While TLR4-NF-κB signaling initially relies on MyD88, TRIF takes over after TLR4 internalization (which will be discussed in the TRIF-dependent TLR4 signaling section). However, TLR3-NF-κB signaling is solely mediated by TRIF. Notably, NF-κB activation induced by internalized TLR4 closely resembles that induced by TLR3. The TRIF-dependent TLR3 signaling pathways are illustrated below (Table 1).

### 2.1 TLR3-TRIF (N-terminal)-IKKε/TBK1-IRF3-IFN-β response

The transcriptional enhancer of the IFN-β promoter plays a crucial role in activating IFN-β expression, which is essential for the immune system’s response to viral infections. It contains four positive regulatory domains (PRD I-IV) that synergistically activate IFN-β expression (Maniatis et al., 1998). Transcription factors that bind to these IFN-β PRD elements include NF-κB, interferon regulatory factor 3 (IRF3), and the heterodimeric transcription factors ATF-2-c-Jun. NF-κB binds to the PRDII site, while IRF3 binds to the PRDIII and PRDI sites (PRDIII-I). ATF-2-c-Jun binds to the PRDIV site (Maniatis et al., 1998). These interactions are vital for initiating the transcription process Fitzgerald et al. demonstrated that IκB kinase epsilon (IKKε) or TBK1 activates PRDIII-I, highlighting the importance of these kinases in IRF3 activation. Their research showed that IRF3 interacts with IKKε and TBK1, leading to its direct phosphorylation and subsequent nuclear translocation—events crucial for initiating the IFN-β response. Additionally, inhibiting IKKε or TBK1 expression through siRNA effectively suppresses IRF3 activation induced by viral infection, dsRNA treatment, or TRIF overexpression (Fitzgerald et al., 2003). This emphasizes their essential role in the TLR3-mediated, TRIF-dependent signaling pathway involving IRF3 activation. Sato and his colleagues (Sato et al., 2003) also documented that TBK1 binds to the N-terminal of TRIF triggering the assembly of a TRIF-TBK1-IRF3 complex. Importantly, this complex formation relies on TBK1’s kinase activity for phosphorylation and subsequent activation of IRF3. This activation ultimately triggers IFN-β transcription, initiating an immune response against pathogens.

### 2.2 TLR3-TRIF (N-terminal)-TRAF6-TAK1-TAB2-NF-κB activation

Following poly (I: C) stimulation of TLR3 in HEK293T cells, Jiang et al. demonstrated TRIF’s crucial role in recruiting TRAF6 downstream of TLR3 (Jiang et al., 2003; Jiang et al., 2004). TRIF interacts with the TRAF domain of TRAF6 through its N-terminal TRAF6 binding motif. Subsequently, TRAF6 catalyzes the synthesis of K63-linked polyubiquitin chains, which bind to the ubiquitin-binding domain of TAB2. The interaction promotes TAK1 autophosphorylation, leading to its activation (Chen, 2012). Upon cytoplasmic phosphorylation and activation of TAK1, two key players IKK and NF-κB are subsequently activated, playing a pivotal role in the cellular response to external stimuli. Notably, dominant-negative mutants of TRAF6 (DN-TRAF6) and TAK1 (DN-TAK1) specifically inhibit TRIF-dependent NF-κB activation but not IRF3 activation, highlighting the critical roles of both TRAF6 and TAK1 in TLR3-TRIF-mediated NF-κB activation. These findings add valuable insight into the intricate signaling pathways within cells. Further supporting the involvement of TRAF6, Cusson-Hermance et al. treated TRAF6-deficient mouse embryonic fibroblasts (MEFs) with poly (I: C). The absence of IκBα phosphorylation, NF-κB activation, and cytokine production in these TRAF6^−/−^ MEFs demonstrated the essential role of TRAF6 in activating these crucial pathways (Cusson-Hermance et al., 2005). Collectively, these findings emphasize the indispensable roles of both TRAF6 and TAK1 in the NF-κB activation process triggered by the TLR3-TRIF signaling pathway. Understanding these intricate processes is crucial for gaining insights into immune system function and developing effective therapeutic interventions.

### 2.3 TLR3-TRIF (C-terminal)-RIPK1- NF-κB activation

Previous research has provided compelling evidence that TRIF activates the NF-κB signaling pathway through at least two distinct pathways: one involving the N-terminal TRAF6 domain and another utilizing the C-terminal region. Studies by Sato and Yamamoto et al. showed that even with N-terminal mutation, TRIF retains its ability to activate NF-κB, suggesting a more prominent role for the C-terminal region (Yamamoto et al., 2002; Sato et al., 2003). However, the precise mechanism remains unclear. RIPK1, known for its role in the innate immune response against dsRNA viruses (Hildebrand and Murphy, 2016; Zhang et al., 2021), is a potential mediator. The C-terminal RHIM domain of TRIF may interact with RIPK1, leading to downstream NF-κB activation. Supporting this, Meylan et al. found that while NF-κB and JNK pathways were activated in RIPK1^+/−^ MEFs upon TLR3 agonist stimulation, NF-κB activation was inhibited in RIPK1^−/−^ MEFs. They also demonstrated RIPK1 binding to the C-terminal RHIM domain of TRIF, followed by polyubiquitination and subsequent formation of the TAK1-TAB2 complex, ultimately promoting TLR3-mediated NF-κB activation (Meylan et al., 2004; Cusson-Hermance et al., 2005). These findings provide valuable insights into the complex molecular interactions and highlight RIPK1’s crucial role in NF-κB activation.

### 2.4 TLR3-TRIF (C-terminal)-RIPK3-NF-κB inhibition

Meylan et al. demonstrated that overexpressing RIPK3 in 293T cells suppressed TRIF-induced NF-κB activation in a dosage-dependent manner. Because RIPK1 shows a higher binding affinity towards RIPK3 than TRIF. RIPK3 likely inhibits TRIF-RIPK1-mediated NF-κB pathway through competitive biding to RIPK1 (Meylan et al., 2004). Similarly, Sun et al. found that RIPK3 interacts with RIPK1 through their RHIM domains. This interaction ultimately leads to the phosphorylation of RIPK1, thereby inhibiting its-dependent NF-κB activation processes. Mutating the RHIM domain of RIPK3 abolished its inhibitory effect on NF-κB activation, further highlighting the crucial role of RIPK3 in this regulatory process (Sun et al., 2002). These findings suggest potential therapeutic targets for inflammatory and autoimmune diseases.

### 2.5 TLR3-TRIF (C-terminal)-RIPK1-FADD-Caspase8-apoptosis signaling

Apoptosis, a precisely regulated form of programmed cell death, is essential for tissue homeostasis and the elimination of damaged or infected cells. This process relies on a cascade of cysteine proteases, known as caspases, activated within the cell (Hengartner, 2000; Bertheloot et al., 2021; Kowalski et al., 2023). Caspase activation leads to the degradation of various cellular proteins, resulting in cell death. Viral dsRNA induces apoptosis in various cell types through distinct mechanisms (Scarim et al., 2001; Yu et al., 2011; Estornes et al., 2012). This is considered a host defense mechanism, eliminating infected cells to inhibit viral replication and prevent further damage. The adaptor TRIF plays a critical role in TLR3-mediated apoptosis signaling. Studies have shown that synthetic dsRNA induces apoptosis in human breast cancer cells (Cama-1) via a TLR3-TRIF-dependent pathway, accompanied by increased expression of caspase 3 and caspase 8, key executioners of apoptosis (Salaun et al., 2006). McAllister et al. revealed that dsRNA activates TLR3-TRIF-Caspase8-dependent apoptosis in small intestinal mucosal epithelial cells *in vivo*. This activation promotes mucosal remodeling and recovery, vital for maintaining gut homeostasis (McAllister et al., 2013). TLR3 does not directly induce apoptosis due to lacking a death domain (DD). Research has demonstrated that the TLR3-TRIF pathway facilitates the assembly of a death-inducing signaling complex (DISC) comprised of RIPK1 and Fas-associated death domain (FADD). This complex promotes caspase8 oligomerization, subsequently triggering apoptosis to aid the immune system in eradicating infected cells (Salaun et al., 2006). Similarly, Yu et al. demonstrated that poly (I: C) activates caspase-dependent apoptosis in human airway epithelial cells (NCI-H292) in a TLR3-TRIF-RIPK1-dependent manner (Yu et al., 2011). This activation leads to the shedding of soluble type 1 TNF receptor (sTNFR1) from NCI-H292 into the intercellular space to elicit an innate immune response.

### 2.6 TLR3-TRIF (C-terminal)-RIPK3-MLKL necroptosis signaling

Cell death, a vital component of cellular homeostasis, eliminates damaged or infected cells to maintain organismal health. Apoptosis and necroptosis are two extensively studied forms of programmed cell death. Apoptosis is highly regulated and is characterized by membrane blebbing, cell shrinkage, and caspase activation. Necroptosis, a more recently characterized form, is triggered by various factors, including caspase inhibition or impairment (Egorshina et al., 2022; Sahoo et al., 2023). Necroptosis relies on RIPK1 and RIPK3 activation, and its morphology differs from apoptosis with organelle swelling and membrane rupture (Vandenabeele et al., 2010; Shan et al., 2018; Bertheloot et al., 2021). RIPK3 is crucial for initiating necroptosis (Zhang et al., 2009; Morgan and Kim, 2022). It forms a necrosome complex with RIPK1, promoting RIPK3 dimerization and self-phosphorylation. This recruits and phosphorylates the downstream effector MLKL. Phosphorylated MLKL forms polymers that induce cell lysis and necroptosis (Wu et al., 2014; Najjar et al., 2016). While essential for clearing damaged cells, dysregulated necroptosis can contribute to pathological conditions. He et al. demonstrated that TLR3 activation by poly (I: C) in mouse bone marrow-derived macrophages (BMDMs) induced TRIF-RIPK3 interaction via their RHIM domains. This complex promoted reactive oxygen species (ROS) accumulation, which, combined with the caspase inhibitor, z-VAD, triggered necroptosis (He et al., 2011). Cook et al. showed that even with RIPK1 silencing in MEFs, poly (I: C) could still induce necroptosis through RIPK3-MLKL interactions leading to the precipitation of MLKL and the subsequent induction of necroptosis (Cook et al., 2014). These two studies, conducted by separate research teams, offer a more comprehensive understanding of the complex cellular processes underlying necroptosis. They not only highlight the importance of TLR3, TRIF, RIPK3, and MLKL in this process but also provide new insights into potential therapeutic interventions targeting these molecules in TLR3-TRIF-mediated necroptosis signaling.

## 3 TRIF-dependent TLR4 signaling

In TLR4 signal transduction, Yamamoto M et al. demonstrated that TRIF participated in the late activation of NF-κB and IRF3. The activation of NF-κB mediated by TLR4 requires the involvement of MyD88 and TRIF, with MyD88 playing an early activation role in this process (Yamamoto et al., 2003). TLR4 plays a pivotal role in mammalian immune responses, being the sole TLR capable of activating both MyD88 and TRIF-dependent signaling pathways. TLR4 recognizes a wide variety of ligands (Kagan et al., 2008). TLR4 forms a complex with MD-2, a secreted glycoprotein that recognizes and binds LPS with the assistance of the GPI-anchored protein CD14 (Park et al., 2009; Ciesielska et al., 2021). TLR4 located on the cell surface triggers the innate immune response via MyD88-dependent signaling pathway (Kagan and Medzhitov, 2006). Subsequently, TLR4 internalizes into the endosomal membrane, predominantly relying on TRIF to initiate the IFN response and transmit signals to antigen-presenting cells, playing a crucial role in defending against viral infections and initiating the subsequently acquired immunity (Satoh and Akira, 2016; Ciaston et al., 2022; Duan et al., 2022). This process requires LPS binding protein (LBP) for TLR4 internalization (Tsukamoto et al., 2018). The TRAM adaptor is present on both the plasma membrane and endosomal membrane, while TIRAP (MyD88 adaptor-like, Mal) is exclusively localized to the cell membrane and not found on the endosomal membrane (Botelho et al., 2000; Bryant et al., 2015). As a result, the internalization of TLR4 does not activate the TIRAP-MyD88 signaling pathway (Kaganet al., 2008). Besides, Kagan et al. also discovered that inhibition of TLR4 internalization through dynasore treatment prevented TRIF-dependent IRF3 phosphorylation, thereby impeding the IFN response. These findings suggest that TRIF-dependent signaling occurs subsequently after TLR4 internalization. TLR4-TRIF activation mediates the initiation of NF-κB signaling pathway, IFN-β response, and cell death signaling pathway (Ma et al., 2005; Kagan et al., 2008; He et al., 2011; Marongiu et al., 2019). The activation of TLR4 triggers MyD88 and TRIF-dependent signaling pathways in a coordinated manner, allowing for a comprehensive immune defense against both bacterial and viral infections from various sources (Husebye et al., 2006). Other TLRs recognize single ligands and can induce sufficient inflammatory cytokines by activating MyD88 or TRIF-dependent signaling pathways (Kawai and Akira, 2010). Additionally, the activation of TRIF-dependent signaling following TLR4 internalization has been found to exert a negative regulatory effect on TLR4 signaling, preventing sustained activation and contributing to the delicate balance of the body’s immune response. This is crucial as an excessive host reaction to LPS can result in life-threatening complications such as septic shock, multiple organ failure, and mortality (Husebye et al., 2006). The TRIF-dependent TLR4 signaling pathways are shown below (Table 1).

### 3.1 TLR4-TRIF (N-terminal)-TRAF3-TBK1-IKKε-IRF3/IRF7-IFN-β response

TRIF plays an essential role in the TLR3/TLR4-mediated IFN-β response, independent of MyD88 signaling (Kawai et al., 2001; Oshiumi et al., 2003; Sato et al., 2003; Yamamoto et al., 2003). Upon activation of the ubiquitin ligase TRAF3, the N-terminal of TRIF interacts with TBK1 and IKKε, setting off a cascade of events. TBK1, a key kinase, phosphorylates the pLxIS motif on TRIF, allowing IRF3 binding. Subsequently, TBK1 phosphorylates IRF3, triggering its dissociation from TRIF. This dissociation is crucial for the formation of a phosphorylated IRF3 dimer, which translocates to the nucleus and activates the IFN-β signaling pathway (Fitzgerald et al., 2003; Liu et al., 2015; Tsukamoto et al., 2018). In addition to its role in IRF3 activation, TBK1 also mediates the phosphorylation of interferon regulatory factor 7 (IRF7), inducing the expression of IFN-β-related genes, further amplifying the immune response (Häcker et al., 2006). A recent study by Tsukamoto et al. (Tsukamoto et al., 2018) demonstrated that LBP regulates LPS-induced internalization of TLR4, promoting TRIF-dependent activation of the TBK1-IKKε-IRF3-IFN-β pathway in macrophages. This research adds depth to our understanding of how TRIF, TBK1, IKKε, IRF3, and IRF7 collaborate to mount an IFN-β response against pathogens.

### 3.2 TLR4-TRIF (N-terminal)-TRAF6-TAK1-IKKs-NF-κB activation

As previously mentioned, in the LPS-induced TLR4 signaling pathway, two key adaptor proteins, MyD88 and TRIF, sequentially activate NF-κB. When TLR4 is activated by LPS, MyD88 rapidly transmits NF-κB signals from the cytoplasmic membrane, while TRIF induces delayed activation of NF-κB signals (Yamamoto et al., 2003). Sato et al. revealed a functional interaction between TRAF6 and TRIF, facilitated by TRAF6’s TRAF domain and the TRAF6 binding motif (PEEMSW, amino acids 250–255) on TRIF’s N-terminal. They showed that TRAF6 associates with TAK1 and TAB2 to activate IKKαβγ complex (IKKs). IKKs activation then resulted in IκBα phosphorylation and polyubiquitination, followed by proteasomal degradation, abolishing its inhibitory function on p65/p50 NF-κB complex (Sato et al., 2003; Bakkar and Guttridge, 2010; Duan et al., 2023). This liberated complex translocates to the nucleus to activate target gene expression. Cusson-Hermance et al. further supported TRAF6’s role in TRIF-dependent TLR4-induced NF-κB activation. Using TRAF6^−/−^ MEFs, they demonstrated that LPS stimulation did not lead to IκBα phosphorylation, NF-κB activation, or cytokine production, highlighting TRAF6’s essentiality in this pathway (Cusson-Hermance et al., 2005). Thus, TRAF6 is critical for late activation of NF-κB by LPS in a manner of TRIF-dependent TAK1 and IKKs activation. Unlike TRIF-dependent NF-κB signaling, MyD88 downstream of TLRs recruits interleukin-1 receptor-associated kinase 4 (IRAK-4) through death domain interactions. This forms the MyD88some complex, promoting IRAK-4 dimerization, self-phosphorylation, and activation. Activated IRAK-4 then phosphorylates IRAK-1, leading to downstream TRAF6 activation. Upon activation, TRAF6 recruits TAK1, initiating a cascade similar to the TRIF(N-terminal)-TRAF6-TAK1-IKKs-NF-κB pathway, ultimately resulting in NF-κB activation (Kawagoe et al., 2008; Lin et al., 2010; Wang et al., 2017; Yadav and Shirumalla, 2023).

### 3.3 TLR4-TRIF (C-terminal)-RIPK1-NF-κB activation

Studies have shown that TRIF downstream of TLRs mediates the formation of a secondary endosomal complex involving RIPK1 and RIPK3 molecules. Najjar et al. demonstrated that RIPK1 is essential for regulating LPS-induced inflammatory gene expression and that TRIF is necessary for RIPK1-dependent expression of these genes. Specifically, RIPK1 binds to the C-terminal of TRIF downstream of TLR4 via its RHIM domain (Najjar et al., 2016). Cusson-Hermance et al. isolated MEFs from RIPK1^−/−^MYD88^−/−^ double-knockout mice and observed no IκB degradation or NF-κB activation upon LPS treatment. This suggests that RIPK1 participates in the late activation of NF-κB mediated by TLR4 in a TRIF-dependent way. Their luciferase reporter assays further revealed that RIPK1 only activates NF-κB, not IRF3-dependent reporter activity (Cusson-Hermance et al., 2005). Najjar et al. also showed that RIPK1 directly binds to ERK1/2 in LPS-stimulated BMDMs, leading to ERK1/2 phosphorylation and subsequent cFos activation. This activates AP-1, ultimately inducing the expression of inflammatory factors (Najjar et al., 2016).

### 3.4 TLR4-TRIF (C-terminal)-RIPK3-NF-κB inhibition

Najjar et al. demonstrated that in MEFs, both genetic knockout and pharmacological inhibition of RIPK3 (using GSK’872) effectively prevented necroptosis induction. However, these interventions did not reduce the expression of inflammatory factors TNF-α and IL-6 within the cells (Najjar et al., 2016). This is interesting, as RIPK3 has been previously reported to participate in the regulation of inflammatory responses. Similar to the TLR3-TRIF (C-terminal)-RIPK3-NF-κB signaling, RIPK3 interacts and phosphorylates RIPK1 through its RHIM domain. This inhibits RIPK1-dependent NF-κB activation, suggesting that RIPK3 has a crucial role in the regulation of immune responses and inflammation. Furthermore, disrupting the RHIM domain in RIPK3 completely abolished its ability to suppress NF-κB activation. This provides further evidence of the importance of this domain in regulating RIPK3 function. Based on these findings, it can be inferred that RIPK3 competitively inhibits TRIF-dependent TLR4-RIPK1-NF-κB signaling pathway through its RHIM domain. This suggests that RIPK3 may be a potential therapeutic target for the treatment of inflammatory conditions, as targeting this pathway could potentially lead to the inhibition of inflammation and the prevention of tissue damage.

### 3.5 TLR4-TRIF (C-terminal)-RIPK1-FADD-Caspase8-apoptosis signaling

Previous studies have demonstrated that inhibiting NF-κB activity initiates apoptosis in macrophages. This process depends on several key proteins, including RIPK1, caspase8, and FADD (Ma et al., 2005). RIPK1, a central player in cellular apoptosis, has three main domains: a kinase domain, an RHIM domain, and a DD (Wu et al., 2014). Blocking caspase3 activation reversed cell death induced by TRIF, indicating that TRIF-mediated cell death is apoptosis in nature. Furthermore, the ability of TRIF to induce apoptosis is closely linked to its C-terminal RHIM domain; mutation or deletion of this domain abolishes the capacity of TRIF to initiate apoptosis. The RHIM domain allows TRIF to bind to RIPK1. Once bound, RIPK1 recruits FADD through its DD domain, activating caspase8 and subsequently inducing apoptosis (Grimm et al., 1996). This process is crucial for eliminating infected or damaged cells, and clearing debris and pathogens from the body. A study conducted by Kaiser and his team (Kaiser and Offermann, 2005) revealed that TRIF overexpression induces apoptosis in 293T cells through activation of the FADD-caspase8 axis, a crucial signaling pathway. This discovery offers valuable insights into the role of TRIF and RIPK1 in cell apoptosis and underscores the significance of these proteins in maintaining cellular homeostasis. The implications of this study are extensive, and it has the potential to pave the way for innovative therapeutic strategies aimed at apoptosis-related diseases in the future.

### 3.6 TLR4-TRIF (C-terminal)-RIPK3-MLKL-necroptosis signaling

TRIF-dependent signaling via TLR3 and TLR4 activates the TRIF-RIPK3 complex, directly triggering RIPK3 kinase-dependent necroptosis. This process is independent of NF-κB, TNF, and RIPK1 (Kaiser et al., 2013). Interestingly, the FADD-caspase8 complex has been shown to impede the activation of RIPK3, suggesting that RIPK3-mediated necroptosis is suppressed in the presence of apoptosis signals. This implies a built-in cellular mechanism to prevent excessive cell death and maintain tissue homeostasis. Research by He et al. showed that LPS-induced TLR4 activation facilitates TRIF-RIPK3 interaction via their RHIM domains. This complex formation promotes ROS accumulation and triggers necroptosis in BMDMs when used in conjunction with the caspase inhibitor z-VAD (He et al., 2011). The use of z-VAD suggests that necroptosis signaling is activated in response to apoptosis inhibition.

### 3.7 TLR4-TRIF (N-terminal)-IRF1-CMPK2/SAMHD1-mtDNA synthesis

A study by Zhong et al. showed that LPS-induced TLR4 activation triggers the transcription of mitochondrial deoxyribonucleotide kinase 2 (CMPK2) in macrophages (Zhong et al., 2018). This process relies on interferon regulatory factor 1 (IRF1), a key transcription factor, and occurs via both MyD88-and TRIF-dependent TLR signaling pathways. CMPK2 transcription plays a pivotal role in providing deoxyribonucleotides necessary for mitochondrial DNA (mtDNA) synthesis (Zhong et al., 2018). This leads to mtDNA replication, production of oxidized mtDNA (ox-mtDNA), and subsequent activation of NLRP3 inflammasome. Furthermore, the study found that TRIF can activate the expression of SAM and HD domain-containing deoxynucleoside triphosphate triphosphohydrolase 1 (SAMHD1). SAMHD1 acts as a negative feedback regulator of mtDNA synthesis and IL-1β production. The comprehensive study by Zhong et al. uncovers the molecular mechanisms behind TRIF-dependent mtDNA synthesis, highlights the importance of IRF1, and emphasizes the role of MyD88 and TRIF in mediating these signaling pathways. Additionally, the identification of SAMHD1 as a negative feedback regulator offers fresh insights into the complex networks controlling mtDNA synthesis and IL-1β production in macrophages.

## 4 Relationship between TRIF and liver diseases

As a critical TLR adaptor, TRIF participates in various signaling pathways and pathological processes (Chow et al., 2006; Woo et al., 2009; Woo et al., 2012). It can activate eukaryotic initiation factor 2B (eIF2B) to delay apoptosis (Woo et al., 2012). Furthermore, TRIF modulates metabolic pathways through its downstream signaling molecule, IRF3, which downregulates retinoid X receptor α (RXRα), influencing the transcription of numerous metabolism-associated genes (Chow et al., 2006). TRIF has been linked to diseases such as atherosclerosis, amyotrophic lateral sclerosis, and herpes simplex encephalitis (Lundberg et al., 2013; Komine et al., 2018; Uyangaa et al., 2023). Further studies have revealed TRIF’s role in liver diseases. Next, we summarized the role of TRIF-dependent signaling pathways in various liver diseases.

### 4.1 Drug-induced liver injury

The liver is a vital organ for metabolic detoxification, frequently subjected to stimulation from various chemical drugs and derivatives derived from plants. The activation of macrophage TLR4-TRIF-NLRP3-caspase1-IL-18 signaling axis has been found to exacerbate liver injury in a mouse model induced by sequential treatment with *Propionibacterium acnes* and LPS (Imamura et al., 2009). In a murine model of monocrotaline-induced hepatic sinusoidal obstruction syndrome (HSOS), Huang et al. discovered that TLR4^−/−^, MyD88^−/−^, and TRIF^−/−^ mice treated with MCT exhibited attenuated liver injury compared to wild-type (WT) mice, as evidenced by decreased levels of serum ALT and AST, decreased expression of liver MMP9 and nuclear p65. They further revealed that MCT treatment induced the release of DAMPs such as HMGB1 and HSP60 in the injured liver, thereby activating the TLR4-MyD88/TRIF-NF-κB signaling pathways and exacerbating HSOS progression (Huang et al., 2019). Ma et al. established an experimental chronic liver injury mouse model by intraperitoneal injection of carbon tetrachloride (CCL4), which resulted in upregulation of liver TLR2, TLR3, TLR4, MyD88, TRIF, and NF-κB. Their findings suggest that CCL4-induced liver injury can be mediated by TLR-dependent signaling pathway (Ma et al., 2023). In a cellular model of acetaminophen (APAP)-induced liver injury, Minsart et al. observed that 10 mM APAP treatment significantly suppressed hepatocyte activity and increased HMGB1 release in the supernatant of HepRG cells for a duration of 24 h. Subsequently, they separately exposed HepRG cells to recombinant human HMGB1 (rhHMGB1) and APAP, which resulted in the inhibition of cell activity and promotion of hepatocyte death through the TRIF-RIPK3-necroptosis signaling pathway *in vitro* (Minsart et al., 2020). Their findings suggest that the TLR4-TRIF-RIPK3 signaling axis plays a crucial role in APAP-induced liver injury. In conclusion, TRIF emerges as a detrimental molecule in drug-induced liver injury.

### 4.2 Ischemia-reperfusion liver injury (IRI)

The standard treatment for liver failure is liver transplantation, during which ischemia-reperfusion injury occurs most frequently. The pathogenesis of IRI involves the damage caused by reactive oxygen species, such as free radicals, and the activation of inflammatory cytokines like TNF-α, IL-1, and IL-6 through innate immune signaling pathways. Mahmoud et al. demonstrated that Limonin ameliorated Wister rat IRI by suppressing hepatic TLR2/TLR4-MyD88/TRIF signaling (Mahmoud et al., 2014). Zhai et al. discovered that in a mouse model of IRI, liver inflammation and hepatocyte injury induced by liver ischemia/reperfusion were mediated through the activation of TLR4-TRIF-IRF3-dependent pathway rather than MyD88-dependent pathway (Zhai et al., 2004). Kang et al. observed upregulation expression of TLR3, TLR4, MyD88, and TRIF proteins in the livers of Sprague-Dawley rats subjected to liver ischemia/reperfusion (Kang et al., 2011; Kang and Lee, 2012). These findings collectively support that TRIF plays a harmful role in IRI injury by activating TRIF-IRF3 and TRIF-NF-κB signaling pathways to exacerbate liver damage.

### 4.3 Autoimmune hepatitis (AIH)

Autoimmune hepatitis (AIH) is a progressive chronic liver disease of unknown etiology, characterized by elevated levels of immunoglobulin G (IgG) and autoantibodies in the blood, as well as increased serum ALT and AST levels. Previous studies have demonstrated the involvement of the TLR4-NF-κB pathway in the inflammatory response underlying AIH (Cai et al., 2022; Ibrahim et al., 2023; Kang et al., 2023). Cai et al. induced an AIH mouse model by intraperitoneal injection of liver S100 antigen. They observed that the expression levels of liver TLR4 and TRIF proteins were significantly elevated compared to control mice. Additionally, they found a significant increase in the mRNA and protein expression levels of liver TNF-α and NF-κB, while the expression levels of I-κB mRNA and protein were decreased. Their findings suggest that liver S100 atigen activates TLR4-TRIF-NF-κB signaling pathway to induce AIH in mice (Cai et al., 2022). However, their study did not investigate the role of TRIF whole-body knockout or hepatocyte-specific TRIF knockout in the AIH mouse models for further validation. Future research should focus on exploring the specific function of hepatocyte TRIF molecule in AIH.

### 4.4 Viral hepatitis

Hepatitis B virus (HBV) is a hepatotropic DNA virus that can cause either transient or chronic liver infection, leading to potentially fatal acute hepatitis, explosive hepatitis, cirrhosis, and hepatocellular carcinoma (HCC) (Guo et al., 2009). HBV infection is determined by a complex interaction between HBV replication and the host immune system (Ma et al., 2017). The activation of the TLR signaling pathway leads to the production of type 1 interferon and inflammatory cytokines, which have been reported to possess inhibitory effects on hepatotropic virus replication. Additionally, TLR plays a crucial role in inducing specific immune responses against HBV infection and promoting HBV clearance. Guo et al. transfected HepG2 and Huh7 cells with pHBV1.3 to construct HBV-infected hepatocytes. They found that overexpression of TRIF in HepG2 and Huh7 cells inhibited HBV replication, which was represented by decreased HBV mRNA and DNA in HepG2 and Huh7 cells transfected with saTRIF plasmid. In addition, they demonstrated that saTRIF overexpression activated the NF-κB signaling pathway in HBV-infected HepG2 and Huh7 cells, thereby playing a crucial role in antiviral defense (Guo et al., 2009). HBV X protein (HBx), the sole nonstructural protein (NS) of HBV, has been discovered to function as a deubiquitinating enzyme, inhibiting IFN production by suppressing IRF3 and IRF7 ubiquitination. Additionally, HBx can deubiquitinate TRAF3 and IKKi to impact IFN production (Jiang and Tang, 2010). Co-immunoprecipitation experiments have also revealed that HBx can interact with TRIF, NEMO, and IRF3 individually, thereby impeding the activation of downstream TLR3-mediated IFN transcription factors and evading the body’s antiviral defense mechanisms. The TLR4-MyD88/TRIF-NF-κB signaling pathway plays distinct roles in different stages of HBV infection, activating appropriate immune responses against HBV replication in the early stage and promoting the progression of HBV cirrhosis or liver cancer when persistent chronic HBV infection (Zare-Bidaki et al., 2014). Similarly, hepatitis C virus (HCV) infection is also a major cause of chronic hepatitis, cirrhosis, and hepatocellular carcinoma. HCV dsRNA can bind TLR3 on endosomal membranes and inhibit HCV replication by recruiting downstream TRIF to activate IFN response (Colasanti et al., 2023). Premashis et al. found that the expression of TLR3 and TRIF in the liver of HCV patients decreased, and the expression of TLR3 and TRIF was closely related to the severity and outcome of HCV disease (Kar et al., 2017). In summary, TLR3-TRIF mediated IFN response plays an important role in immune surveillance against HBV and HCV infection.

### 4.5 Alcoholic liver disease (ALD)

In ALD, long-term alcohol consumption results in the impairment of intestinal barrier function, leading to the translocation of gut-derived lipopolysaccharide (LPS) to the liver and subsequent activation of the liver TLR4 signaling pathway, thereby mediating the occurrence and progression of ALD (Chen et al., 2023; Chen et al., 2023; Shibamoto et al., 2023). Zhuang et al. demonstrated that ginger-derived nanoparticles (GDNs) exert antioxidant and anti-inflammatory effects in mouse primary hepatocytes by promoting the nuclear translocation of Nrf2 through TLR4-TRIF signaling pathway. Furthermore, their study revealed the potential of GDNs in ameliorating liver steatosis and inflammation in ALD. However, it remains to be investigated whether the beneficial effects of GDNs on ALD are mediated through activation of the TLR4-TRIF-NRF2 axis, warranting further investigation (Zhuang et al., 2015). In a 4-week chronic alcohol-feeding ALD mice model, Petrasek et al. discovered that the activation of the TLR4-TRIF-IRF3 signaling pathway in hepatic parenchymal cells induces a type 1 IFN response, which plays an anti-inflammatory role and improves the progression of ALD disease (Petrasek et al., 2011). Additionally, they observed that IRF3 whole-body knockout ameliorated liver injury in ALD mice by alleviating liver steatosis and inflammation. These findings suggest that the TRIF-IRF3 signaling axis may exhibit distinct roles in the pathogenesis of liver diseases depending on cell type and tissue microenvironment. Meanwhile, Hritz et al. found that chronic alcohol feeding exacerbated alcoholic liver injury through the activation of TLR4-TRIF-NF-κB or TLR4-TRIF-IRF3/IRF7 signaling pathways (Hritz et al., 2008). They found that chronic alcohol-fed TLR4 knockout mice showed reduced liver damage, including less hepatic steatosis, lower levels of liver enzymes, and decreased expression of liver TNF-α, IL-6, IRF3, and IRF7 compared to WT, TLR2 knockout, and MyD88 knockout mice. Their study suggests that chronic alcohol consumption exacerbates the progression of ALD through a TLR4-MyD88-independent pathway. After 4 weeks of chronic alcohol feeding, WT and TRIF knockout female mice were intraperitoneally injected with LPS (1.0 μg/g). Compared to WT mice, TRIF knockout mice exhibited decreased serum levels of TNF-α and ALT, as well as ameliorative liver steatosis. These findings indicate that the combination of chronic alcohol feeding and intraperitoneal injection of LPS exacerbates liver injury in ALD mice by inducing abnormal TNF-α expression through TLR4-TRIF signaling pathway activation (Zhao et al., 2008). Overall, TRIF molecule has a dual role in ALD, with its regulatory effects on disease progression varying across different tissues and cells through distinct signaling pathways.

### 4.6 MASLD/MASH

Metabolic dysfunction-associated steatotic liver disease (MASLD), previously known as Metabolic associated fatty liver disease (MAFLD) and Nonalcoholic fatty liver disease (NAFLD), is the leading cause of chronic liver disease globally and can progress to Metabolic dysfunction-associated steatohepatitis (MASH), hepatic fibrosis, cirrhosis, and even hepatocellular carcinoma (Younossi et al., 2016; Quek et al., 2023; Rinella et al., 2023; Vaz et al., 2023). In a 10-week HFD-induced MASLD mouse model, TRIF knockout mice exhibited aggravating liver steatosis and inflammation levels compared to WT mice. This study demonstrated that TLR3-TRIF-IRF3 signaling suppressed stearoyl–coenzyme A desaturase 1 (SCD1) transcription and subsequent lipid synthesis (Chen et al., 2017), suggesting the protective role of TRIF. Our previous study found that in a 22-week choline-deficient amino acid (CDAA) diet-induced MASH mouse model, TRIF knockout exacerbated liver inflammation in MASH mice by inducing hepatocytes and Kupffer cells to produce CCL3 and CXCL1, recruiting macrophages and neutrophils, while simultaneously alleviating liver steatosis through downregulation of DGAT2 expression in hepatocytes. This study suggests that TRIF can promote liver steatosis and inhibit liver inflammation in MASH(Yang et al., 2017). We observed that previous studies investigating the role of TRIF in MASLD predominantly employed macrophage-specific TRIF knockout or whole-body TRIF knockout mice. However, the role of TRIF in the progression of MASLD is still unclear in hepatocytes, which are the main metabolic cells. In addition, whether hepatocyte TRIF is a protective or detrimental molecule has not yet been determined, which requires further study in the future.

Notably, the protective or detrimental nature of TRIF has not yet been determined, it exerts different effects depending on disease stage, disease model, type of cells studied, and upstream or downstream signaling molecules and pathways involved. In the following sections, we will discuss TRIF’s dual roles in liver steatosis, inflammation, fibrosis, and carcinogenesis within the context of different liver diseases and disorders.

## 5 The role of TRIF in liver disorders

### 5.1 TRIF and liver steatosis

In a CDAA diet-induced MASH mouse model, TRIF-deficient mice exhibited less severe liver steatosis compared to wild-type mice (Yang et al., 2017). This finding was supported by *in vitro* experiments, where TRIF-deficient and TLR4-deficient hepatocytes displayed resistance to lipid accumulation induced by palmitic acid and LPS, unlike wild-type cells (Yang et al., 2017). These studies suggest that TRIF promotes liver steatosis under certain conditions. Additionally, DGAT2, a downstream effector, was identified as a potential contributor to lipid accumulation in hepatocytes through the TLR4-TRIF pathway stimulated by free fatty acids (Yang et al., 2017). Similarly, TRIF deficiency in an alcohol-associated liver disease (ALD) animal model also led to a reduction in liver steatosis (Zhao et al., 2008). These findings suggest that TRIF functions as a facilitator of liver steatosis under certain conditions. However, another study revealed a protective effect of TRIF on hepatic steatosis under HFD conditions (Chen et al., 2017). TRIF-deficient mice on HFD displayed exacerbated liver steatosis potentially due to up-regulating expression of SCD1, a rate-limiting enzyme for lipogenesis. In this case, TRIF activation led to IRF3 binding to the Scd1 promoter region, inhibiting its transcription and preventing lipogenesis (Chen et al., 2017). These findings collectively demonstrate that TRIF plays diverse roles in liver steatosis, and these variations can be attributed, at least in part, to the specific signaling pathways triggered by different stimuli.

### 5.2 TRIF and liver inflammation

The TLRs-MyD88-NF-κB signaling pathway is well-established as an inflammation-related pathway crucial in various pathological processes (Medzhitov, 2001; Woo et al., 2009; Cole et al., 2011; Jia et al., 2014). TRIF-dependent pathways, acting as essential downstream elements of TLRs, also participate in modulating inflammatory responses. TRIF-deficient mice exhibit resistance to the upregulation of inflammatory cytokines like IL-6 and IL-1β induced by various stimuli. In an IRI mouse model, TRIF, rather than MyD88, was found to be responsible for inflammation propagation (Zhai et al., 2004). Furthermore, TRIF depletion offers protection against inflammatory insults induced by ischemia-reperfusion (Zhai et al., 2004; Brempelis et al., 2017). Beyond IRI, the TRIF-IRF3 signaling axis also impacts the inflammatory progression of ALD (Zhao et al., 2008). The dysregulation of TNF-α in macrophages induced by chronic alcohol exposure highly depends on the TRIF-IRF3 pathway (Zhao et al., 2008). In a mouse model of AIH, it was found that TLR4 sequentially activates TRIF and NF-κB, thus accelerating inflammatory responses (Cai et al., 2022). In these aforementioned models, TRIF acts as a promoter of liver inflammation. However, in a MASH mouse model fed with CDAA diet, TRIF plays a protective role. Compared with wild-type mice fed with CDAA diet, TRIF-deficient mice exhibited exacerbated liver inflammation, evidenced by increased hepatocyte ballooning, higher levels of TNF levels, and elevated alanine aminotransferase levels (Yang et al., 2017). The worsened inflammation observed due to TRIF deficiency might be partly attributed to the increased chemokines production, which can recruit monocytes and macrophages, amplifying inflammatory cascades (Yang et al., 2017). Additionally, TRIF restricts MASH inflammation through IFN-β secretion modulation. This signaling pathway, primarily regulated by TLR4-TRIF-IRF3, plays a role in innate immunity (Hidmark et al., 2005). In MASH, TLR4-TRIF-IRF3 activation enhanced IFN-β secretion, stimulating macrophage activation, NK cell cytotoxicity, B cell differentiation, and proliferation, while inhibiting Th1 cell activation and proinflammatory cytokine expression. This ultimately impedes the pathological progression of MASH (Hidmark et al., 2005).

### 5.3 TRIF and liver fibrosis and HCC

TRIF’s influence extends beyond liver steatosis and inflammation, also impacting liver fibrosis. In a CDAA-induced MASH mouse model, Yang et al. demonstrated that TRIF depletion worsens liver fibrosis (Yang et al., 2017). This suggests a protective role for TRIF against fibrosis. Its regulatory effect is achieved by modulating the fibrogenic response. TRIF deficiency can lead to increased expression of tissue inhibitor of metalloproteinase 1 (Timp-1) and decreased expression of BMP and activin membrane-bound inhibitor (Bambi), promoting fibrosis development (Seki et al., 2007). Furthermore, TRIF-mediated IFN-β production is a crucial antifibrotic mechanism. IFN-β inhibits hepatic stellate cell activation and suppresses α-smooth muscle actin (SMA) expression (Shimozono et al., 2015). In contrast, TRIF activation appears to promote HCC. The TRIF-NF-κB pathway reportedly increases PD-1 and PD-L2 expression while suppressing IFN-γ and TNF-α, key anti-cancer cytokines. TRIF activation might facilitate HCC development by suppressing host anti-tumor immunity, allowing tumor cells to escape immune surveillance (Xu et al., 2015). These findings have significant clinical implications. The detrimental effects of TRIF activation highlight the need to understand and target the TRIF-NF-κB axis to prevent and treat HCC. Researchers may develop drugs that target TRIF to inhibit or potentially reverse liver fibrosis progression. Additionally, these results underscore the intricate interplay among diverse signaling pathways in regulating liver fibrosis and HCC. The role of TRIF in liver disorders can be referenced to (Table 2).

**TABLE 2:** The role of TRIF in liver disorders.

Reference	Disease model	Effects of TRIF on liver disorders				Potential mechanisms
		Liver steatosis	Liver inflammation	Liver fibrosis	HCC	
Yang et al. (2017)	MASH	Promotion	Inhibition	Inhibition		Inhibiting liver inflammation by reducing the production of chemokines and modulating the secretion of interferon-β; Inhibiting liver fibrosis by downregulating Timp-1 and upregulating Bambi, and modulating the secretion of interferon-β
Zhai et al. (2004)	Liver IRI		Promotion			IFN-β response and IFN-inducible gene production
Zhao et al. (2008)	ALD	Promotion	Promotion			TRIF-IRF3 pathway
Chen et al. (2017)	Fatty liver	Inhibition				Inhibiting liver steatosis via the suppression of Scd1 transcription
Cai et al. (2022)	AIH		Promotion			TLR4-TRIF-NF-κB pathway
Xu et al. (2015)	HCC				Promotion	Enhancing the expression of PD-1 and PD-L2 via TRIF-NF-κB pathway

Abbreviations: HCC, hepatocellular carcinoma; MASH, metabolic dysfunction-associated steatohepatitis; IRI, ischemia-reperfusion injury; ALD, alcoholic liver disease; AIH, autoimmune hepatitis.

As indicated above, TRIF plays a significant role in liver steatosis, inflammation, fibrosis, and carcinogenesis. However, It remains elusive whether targeting TRIF can be an effective method for the treatment of liver diseases in clinical practice. The primary challenge lies in the fact that current investigations into the role of TRIF in various liver diseases predominantly rely on animal or cellular models, and these studies have not been effectively translated to human subjects. A major concern is the significant inter-species differences in TLR upstream of TRIF (Kaur et al., 2022). Besides TRIF, the effects of the molecules implicated in TRIF-associated pathways on liver disease have also seldom been evaluated in clinical trials except for TLR4. A randomized controlled trial by Lu et al. demonstrated that Babao Dan, which inhibits the TLR4 inflammatory pathway, improved neurocognitive function in patients with minimal hepatic encephalopathy (Lu et al., 2021). Despite the lack of clinical research directly targeting TRIF-associated pathways for liver diseases, preclinical studies provide compelling evidence for TRIF’s potential as a therapeutic target. Specifically, activating or inhibiting TRIF depending on the specific liver disease state may restrict the progression of various conditions, including autoimmune hepatitis, alcoholic liver disease, and metabolic dysfunction-associated steatohepatitis. Therefore, developing TRIF-targeting drugs, such as agonists and inhibitors, holds significant promise for treating liver diseases. In addition, TRIF plays distinct regulatory roles in various liver diseases and different disease courses within the same liver disease. While TLR3/TLR4-TRIF activation drives the progression of drug-induced liver injury, ischemia-reperfusion liver injury, and autoimmune hepatitis, TRIF exhibits a dual role in ALD and MASLD/MASH. Furthermore, TLR3/TLR4-TRIF exerts a crucial antiviral role in the initial stage of viral hepatitis, and when viral hepatitis progresses to serious complications such as cirrhosis and hepatocellular carcinoma, TLR3/TLR4-TRIF has been found to accelerate the disease process. Therefore, the timing and risk of targeted TRIF therapy are difficult to control, and excessive activation or suppression of TRIF expression will bring an inestimable risk of autoimmune activation or immunosuppression. Therefore, extensive clinical trials are still necessary to evaluate the safety and efficacy of these novel TRIF regulators in the context of liver disease management.

## 6 Conclusion and perspectives

This comprehensive review summarized the TLR3/TLR4-TRIF signaling pathway, highlighting its intricate association with liver diseases. We explored the pivotal role of TRIF in hepatic pathologies encompassing steatosis, inflammation, fibrosis, and carcinogenesis, suggesting its potential as a promising therapeutic target for future research.

**FIGURE 2:**
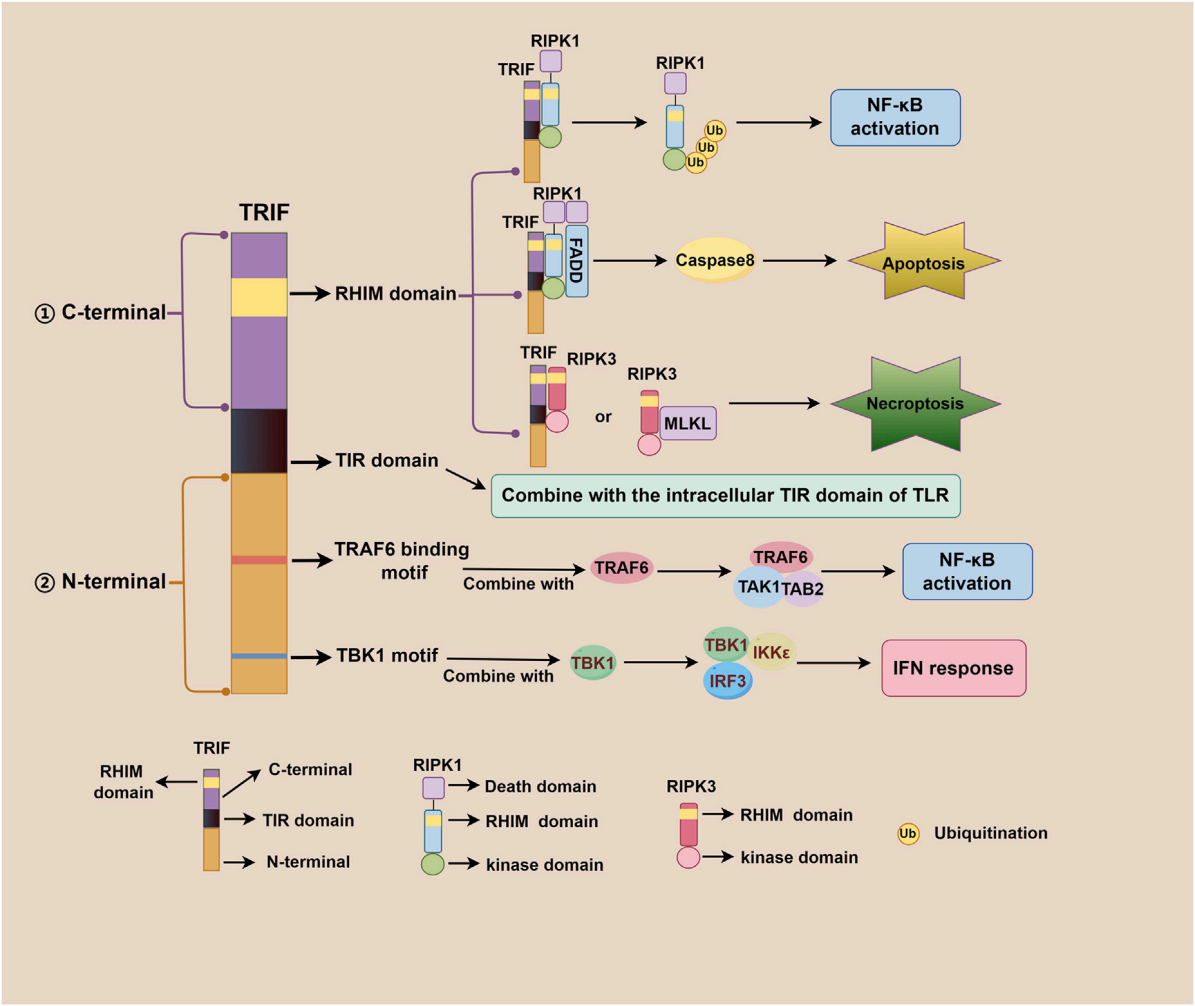
Different domains of TRIF and their corresponding functions. The TRIF molecule consists of a TIR domain flanked by an N-terminal extension and a C-terminal extension. The C-terminal RHIM domain is involved in regulating NF-κB activation, apoptosis, and necroptosis; The TIR domain is responsible for binding to the intracellular TIR domain of TLR; The N-terminal TRAF6 binding motif plays a role in NF-κB activation, and the N-terminal TBK1 motif contributes to IFN response regulation.

TRIF, an adaptor downstream of TLR3 and TLR4, plays a pivotal role in the innate immune response, acquired immune response, as well as cell death signaling pathway (Meylan et al., 2004; He et al., 2011). The TRIF molecule consists of a TIR domain flanked by an N-terminal extension and a C-terminal extension that contains the RHIM domain. Both TRIF-dependent TLR3 and TLR4 signaling pathways share similarities but differ mainly in their recognized ligands. After TLR3 and TLR4 activation, the N-terminal of TRIF interacts with TBK1 and IKKε, leading to IRF3 phosphorylation. Subsequently, phosphorylated IRF3 translocates to the nucleus to initiate the IFN-β response (Fitzgerald et al., 2003; Sato et al., 2003). Additionally, the N-terminal of TRIF also recruits TAK1 and TAB2 through TRAF6, culminating in IκBα phosphorylation and NF-κB activation (Jiang et al., 2003; Jiang et al., 2004). The C-terminal RHIM domain of TRIF further regulates NF-κB signaling through interactions with RIPK1 or RIPK3, with RIPK1 promoting and RIPK3 inhibiting activation (Sato et al., 2003; Meylan et al., 2004). Beyond IFN-β response and NF-κB signaling, TRIF also participates in regulating cell death signals via its C-terminal RHIM domain. TRIF initiates apoptosis signals through the RIPK1-FADD-caspase8 signaling axis and necroptosis signals through the TRIF-RIPK3 complex (He et al., 2011; Yu et al., 2011; McAllister et al., 2013; Cook et al., 2014; Wu et al., 2014). Notably, TRIF downstream of TLR4 can also regulate mitochondrial DNA synthesis and replication by activating transcription factors CMPK2 and SAMHD1 (Zhong et al., 2018). Figure 1 illustrates the complexity and multifaceted nature of TRIF-dependent TLR3/TLR4 signaling pathway, involving diverse signaling molecules and pathways that work together to generate robust immune response and regulate cell death. Figure 2 depicts different domains of TRIF and their corresponding functions. While the intricate relationships between TRIF, TLR3, TLR4, and downstream pathways are still being investigated, ongoing research holds promise for a comprehensive understanding of these mechanisms.

In recent years, an increasing number of studies have reported the pivotal role of TRIF in the pathogenesis of liver diseases (Cai et al., 2009; Riad et al., 2011; Ayoobi et al., 2013; Chen et al., 2017; Cai et al., 2022). Interestingly, TRIF exhibits diverse functions in various liver conditions, impacting both steatosis and inflammation. In steatosis, TRIF can promote lipid accumulation in hepatocytes treated with palmitic acid by upregulating DGAT2 expression. Additionally, it exacerbates hepatic steatosis in ALD mice through the TRIF-IRF3-IFN-β pathway (Zhao et al., 2008; Yang et al., 2017). However, TRIF knockout aggravates hepatic steatosis in a HFD-fed mouse model by enhancing SCD1 transcription and promoting lipid synthesis, suggesting a context-dependent inhibitory role in this model (Chen et al., 2017). Similarly, TRIF can facilitate liver inflammation progression through IRF3 and NF-κB signaling pathways in ALD and AIH models, respectively (Zhao et al., 2008; Cai et al., 2022). Furthermore, TRIF knockout in a hepatic IRI model resulted in reduced expression of inflammatory factors such as IL-6 and IL-1β in the liver (Zhai et al., 2004). These studies suggest that TRIF acts as a detrimental molecule exacerbating liver inflammation. However, TRIF also exhibits a protective role in liver inflammation. Yang et al. demonstrated that TRIF knockout enhances liver inflammation in MASH mice by inducing chemokine production and regulating IFN-β secretion. Additionally, TRIF knockout promotes liver fibrosis by up-regulating Timp-1 expression (Yang et al., 2017). In liver cancer, TRIF-NF-κB pathway activation induces PD-1 and PD-L2 expression, suppressing IFN-γ and TNF-α production in tumor tissue, thereby facilitating hepatocellular carcinoma progression (Xu et al., 2015). The impact of TRIF on hepatic steatosis, inflammation, fibrosis, and carcinogenesis varies across different disease states due to variations in disease models, cell types examined, as well as upstream and downstream signaling molecules involved. Consequently, further comprehensive investigations are warranted to elucidate the precise role played by TRIF in various liver diseases.

Given TRIF’s crucial role in TLR signaling and its diverse effects on liver diseases, future investigations should focus on exploring small molecule inhibitors or agonists targeting TRIF and its associated pathways in specific disease contexts. This holds great promise for the development of novel therapeutic strategies for liver diseases.
